# Upper limb musculoskeletal abnormalities in type 2 diabetic patients in low socioeconomic strata in Pakistan

**DOI:** 10.1186/1756-0500-6-16

**Published:** 2013-01-17

**Authors:** Saera Suhail Kidwai, Lubna Wahid, Shaista A Siddiqi, Rashid M khan, Ishaq Ghauri, Ishaque Sheikh

**Affiliations:** 1 Jinnah Medical College Hospital, SR-6, Sector 7-A, Korangi Industrial Area, Karachi, 75440, Pakistan; 2 MS (neuropharmacology), Karachi, Pakistan

**Keywords:** Type 2 Diabètes mellitus, Adhesive capsulitis, Carpal tunnel syndrome, Dupuytren’s contracture, Trigger finger, Pakistan

## Abstract

**Background:**

Musculoskeletal manifestations of diabetes in the upper limb are well recognized. No data has been available in this regard from Pakistan. Our aim was to find out the frequency of upper limb musculoskeletal abnormalities in diabetic patients.

**Methods:**

This was an observational study in which type 2 diabetes patients attending our diabetic clinic were enrolled along with age and gender matched controls. Data was analyzed on SPSS 16.

**Results:**

In total, 210 Type 2 diabetics (male 34.3%, female 65.7%) and 203 controls (male 35%, female 65%) were recruited. The mean age was 50.7± 10.2 years in diabetic group as compared to 49.5±10.6 years in the control group. The frequencies of hand region abnormalities were significantly higher in the diabetic subjects as compared to the controls (20.4%, p-value <0.001). Limited joint mobility (9.5% vs 2.5%), carpal tunnel syndrome (9% vs 2%), trigger finger (3.8% vs 0.5%), and dupuytren’s contracture (1% vs 0%) were found more frequent as compared to controls (all p-values <0.05). In the shoulder region of diabetic subjects, adhesive capsulitis and tendonitis was found in 10.9% and 9.5% respectively as compared to 2.5% and 2% in control group [p- value <0.001]. A weak but positive relationship was observed between age and duration of diabetes with these upper limb abnormalities. However, no correlation was found between the frequencies of these abnormalities with control of diabetes.

**Conclusion:**

A higher frequency of upper limb musculoskeletal abnormalities was observed in Type 2 diabetic patients as compared to control group.

## Background

Diabetes mellitus (DM) is considered as an epidemic in the modern world and much of its morbidity and mortality is related to micro and macro vascular complications. However, it is also associated with musculoskeletal disorders of the hand and shoulder that can be very incapacitating and significantly compromise their quality of life [1,2].

The manifestations of DM in the hand have been widely discussed in the last two decades, but the exact prevalence is still unknown. There is evidence that these entities are not only more frequent in patients with DM but may also be associated with its duration, poor metabolic control and presence of micro vascular complications [2-5]. Currently, DM affects 240 million people worldwide and this number is projected to increase to 380 million by 2025. Alarmingly, 80% of this burden will affect the low and middle income countries [6]. With 6.9 million known diabetic patients, Pakistan ranks 7^th^ worldwide, and these figures are expected to double by 2025 moving Pakistan to 4^th^ position and affecting 11.5 million people [7-9]. Considering such a high prevalence of DM in our region and its strong correlation with musculoskeletal abnormalities worldwide, it is important to know the magnitude of this relationship in our local population and also the factors which are associated.

Epidemiologic studies have identified several personal, occupational and psychosocial factors related to upper-extremity musculoskeletal disorders [10]. These factors are additive in the presence of DM and thus increase the frequency of hand and shoulder problems in the diabetic population. The exact patho physiology of most of these musculoskeletal disorders remains obscure, however, connective tissue disorders, neuropathy or vasculopathy may have a synergistic effect on the increased incidence of musculoskeletal disorders in DM [11].

According to Crispin and Alcocer, prolonged hyperglycemia in uncontrolled diabetic patients results in collagen glycosylation. Glycosylated collagen is less soluble, offers increased resistance to collagenases and accumulates in connective tissue, which not only alters the extra cellular matrix structure and function but also affects cell viability [3].

Most published studies report that capsulitis and tendonitis in the shoulder are the most common conditions, where as carpal tunnel syndrome (CTS), Dupuytren’s contracture (DCT), trigger finger and limited joint mobility (LJM) are the commonest abnormalities of the hand observed in the diabetic patients [12]. The association of upper limb locomotor disability with uncontrolled diabetes or with its complications like neuropathy, nephropathy or retinopathy have been identified in some studies [2,4,5,13] but not in others [14,15].

Most published international studies include data from USA [16], Europe UK [17], Poland [18], Greece [19] and Australia [20], but there is very limited data from the Asian population [21]. However, the results cannot be directly compared because of the heterogeneity in study population, design and sample size. The aim of our study was to identify the frequency of upper limb musculoskeletal abnormalities in Type 2 diabetes mellitus belonging to a low socioeconomic stratum in Karachi,Pakistan. The possible association of these abnormalities with diabetic complications (e.g. neuropathy, retinopathy and nephropathy) and the relationship of poor glycemic control with musculoskeletal abnormalities in these patients was identified as a secondary objective.

## Methods

This observational study was conducted in Jinnah Medical College Hospital, a tertiary care hospital located in a low socioeconomic area which represents a diverse population comprising of several different ethnic groups in the area. The ethical approval was obtained from the hospital ethical research committee. Two hundred and ten consecutive patients of Type 2 diabetes attending the diabetic clinic were enrolled after informed consent. Demographic data regarding the age, sex, Body Mass Index (BMI), type and duration of diabetes was noted. Exclusion criteria included chronic liver disease, hypothyroidism and history of alcoholism. Ophthalmological examination was done. The presence of retinopathy, neuropathy and nephropathy was also noted. The most recent HbAIc was used to categorize subjects in three groups: Group 1 had ≤ 7%, Group 2 had 7.1-8.4% and Group 3 had ≥ 8.5% [22].

Nephropathy was considered positive if urinary albumin was greater than 30 mg/d. For assessment of diabetic peripheral neuropathy, testing for pain perception was done by a sterile pin, touch by cotton and monofilament testing, vibration by using 128 Hz tuning fork along with propioception [23].

In total, 203 age and sex matched patients were recruited as controls attending the medical out patient department for medical conditions other than a rheumatological complaint.

All the cases as well as controls were surveyed for the presence of symptoms related to upper limb musculoskeletal abnormalities. GALS (gait, arm, legs, spine) screening was performed which if significant lead to REMS (Regional examination for musculoskeletal system) and the following abnormalities were noted. (1) Adhesive capsultis was defined as history of unilateral and/or bilateral pain in the deltoid area with no history of trauma and equal restriction of active and passive glenohumeral movement in a capsular pattern (external rotation > abduction > internal rotation) [24]. (2) Tendonitis was considered positive with presence of inflammation in either of the two tendons i.e. rotator cuff or biceps with a history of pain in the deltoid or anterior shoulder region and restricted active movements in the corresponding muscles [24]. (3) Flexor tenosynovitis or trigger finger was diagnosed by palpating a nodule or thickened flexor tendon with locking phenomenon during flexion or extension of any finger (4) DCT was defined as palpable thickening of palmer fascia with flexor deformity of 2^nd^, 3^rd^,4^th^ and 5^th^ finger. (5) CTS was considered positive with positive Tinel’s/Phalen’s sign, loss of power of abduction pollicis brevis, thenar wasting and reduced sensation in 1^st^, 2^nd^ and 3^rd^ finger that is in the distribution of the median nerve. (6) LJM or diabetic cherioarthropathy was characterized by thick, tight waxy fingers over the dorsal aspect of hands with flexion deformity of metacarpophalangial and interphalangeal joint and inability to make a fist or inability to bring the palms completely together with wrist maximally flexed forming the prayer sign.

SPSS version 16 was used for analysis. Descriptive statistics obtained were reported as mean and proportion with standard deviation (SD) wherever appropriate. Univariate association between diabetic and control groups was compared using Chi square test for categorical data and *t*-test for the continuous variables.

## Result

Overall 210 diabetic patients were recruited in this study. In the diabetic group, there were 72 males (34.3%) and 138 females (65.7%) with a mean age of 50.7 years (SD ± 10.2). BMI in the diabetic cohort was found to be 27.6(SD±5.2). We found that 114(54.28%) diabetic patients had positive GALS screening examination with REM showing positive rheumatological findings in 28 (13.3%) patients with hand, 26 (12.3%) patients with shoulder and 15 (7.14%) patients of both hands and shoulder involvement. The controls group of 203 subjects had 71 males (35%) and 132 females (65%) with a mean age of 49.5(SD±10.6), only 67 patients (33%) had positive GALS examination with 7 (3.45%) having hand involvement, 6 (2.96%) with shoulder abnormality and 3 (1.48%) having both with a significant P value of <0.001 when cases and controls were compared.

In diabetic subjects the most common abnormality in the hand was shared equally among LJM (9.5%) and CTS (9%) followed by trigger finger (3.8%) and DCT (1%). Shoulder abnormalities were more frequent than hand abnormalities with capsulitis affecting 23 (10.9%) and tendonitis 20(9.5%) diabetic patients. The comparison of demographic data and frequencies of upper limb involvement in diabetics and controls are shown in Table 1, and the comparison according to gender is mentioned in Table 2.

**Table 1:** Comparison of demographic data and frequencies of upper limb involvement in diabetics and nondiabetics

	**Study group**		**p-value**
	**Non diabetics**	**Diabetics**	
	**n(%)**	**n(%)**	
**N**	**203**	**210**	
Age (years), mean (SD)	49.5 (±10.6)	50.7 (±10.2)	0.248
**Gender**			
Male	71(35.0)	72(34.3)	0.883
Female	132(65.0)	138(65.7)	
**Duration of Diabetes (years) mean (±SD)**	*NA	6.3(±5.7)	-
**Any Hand Abnormality**	10 (4.9)	43(20.5)	< 0.001
**Any Shoulder Abnormality**	9 (4.4)	41(19.5)	< 0.001
**Any Upper limb Abnormality**	16 (7.9)	69(32.9)	< 0.001
**Trigger Finger**	1 (0.5)	8(3.8)	0.037
**Dupuytrens Contracture**	0	2(1.0)	
**Carpal Tunnel Syndrome**	4 (2.0)	19(9.0)	0.002
**Limited Joint Mobility**	5 (2.5)	20(9.5)	0.003
**Adhesive capsulitis of shoulder**	5 (2.5)	23(11.0)	0.001
**Tendonitis**	4 (2.0)	20(9.5)	0.001
**GALS screen**	67(33.0)	114(54.3)	< 0.001

*NA = not applicable.

**Table 2:** Comparison of Hand and Shoulder abnormalities according to gender

	**Control n (%)**		**Diabetics (MSK) n (%)**	
	**Male**	**Female**	**Male**	**Female**
**N**	**71**	**132**	**72**	**138**
**Any Hand Abnormality**	**1 (10.0)**	**9 (90.0)**	**8 (18.6)**	**35 (81.4)**
Trigger Finger	*NA	1 (100)	1 (12.5)	7 (87.5)
Duputryens Contracture	*NA	*NA	0 (0)	2 (100)
Carpal Tunnel Syndrome	0 (0)	4 (100)	4 (21.1)	15 (78.9)
Limited Joint Mobility	1 (20.0)	4 (80.0)	3 (15.0)	17 (85.0)
**Any Shoulder Abnormality**	**2 (22.2)**	**7 (77.8)**	**14 (34.1)**	**27 (65.9)**
Capsulitis	1 (20.0)	4 (80.0)	6 (26.1)	17 (73.9)
Tendonitis	1 (25.0)	3 (75.0)	8 (40.0)	12 (60.0)
**Hand/Shoulder Abnormality**	**2 (12.5)**	**14 (87.5)**	**20 (29.0)**	**49 (71.0)**

*NA = not applicable.

When the frequency of upper limb musculoskeletal abnormalities is identified categorically in the 3 subgroups of glycemic levels, it is noted that although diabetic patients have a high frequency of upper limb abnormalities compared to controls, but increasing levels of HbA1c do not show increasing correlation with the occurrence of these rheumatological problems . Data was also analyzed for the presence of any correlation between the presence of complications of diabetes (neuropathy, retinopathy and nephropathy) with hand or shoulder involvement which revealed that 13.7% of our diabetic cohort having nephropathy, also had CTS (p-value; 0.05). Similarly, 5% of these had adhesive capsulitis (p-value; 0.02), however, the rest of the musculoskeletal abnormalities had no significant association with this complication. In those with retinopathy, only CTS showed a positive correlation (p-value; 0.05). In diabetic patients having retinopathy, 16.6% also suffered from CTS. Considering diabetic patients having neuropathy, the only significant relationship was found with adhesive capsulitis (p-value; 0.02) where 7.1% of these patients had evidence of adhesive capsulitis.

Furthermore, shoulder and hand abnormalities per se showed a positive correlation (p-value; <0.001) with increasing age and duration of diabetes in this study. Table 3 shows a summary of the data of present study along with some of the other studies done in this regard.

**Table 3:** Overview of international data on upper limb musculoskeletal abnormalities

	**Britain**	**Australia**	**America**	**India**	**Turkey**	**Pakistan**
	**Ramchurn et al. [**[17]**](2009)**	**Smith et al. [**[20]**](2003)**	**Cagliero et al. [**[16]**](2002)**	**Sarkar et al. [**[21]**](2008)**	**Ardic et al. [**[15]**](2003)**	**Saera et al. (2011)**
	**n=96**	**Prevalence**	**n = 100**	**n= 80**	**n=78**	**n=210**
Adhesive Capsulitis	25%	11-30%	12%	23.7%	12.8%	11%
Tendonitis	5%	*NA	*NA	*NA	*NA	9.5%
Limited joint mobility	28%	8-50%	*NA	20%	0	9.5%
Trigger finger	29%	11%	3%	5%	3.8%	3.8%
Carpal tunnel syndrome	20%	11-16%	12%	3.7%	1.3%	9%
Dupuytren’s contracture	13%	20-63%	16%	28.7%	21.8%	1%

*NA = not available.

## Discussion

In this study, we have observed a significant association of hand and shoulder involvement with Type 2 diabetes. We found a weak but positive relationship between advancing age and duration of diabetes with upper limb musculoskeletal abnormalities in our diabetic subjects in accordance with international data [25].No association was found between upper limb musculoskeletal abnormalities and degree of hyperglycemia. However, considering the complications of diabetes, presence of any one of these was only established with CTS and/or adhesive capsulitis. This is an interesting observation since many studies found a strong correlation between these variables [4,5,20,21] while it is refuted by others [11,14-16].

Another important observation was that the frequencies of upper limb musculoskeletal abnormalities in our diabetic subjects were found to be significantly lower than the international or regional data [21] as shown in Table 3. It may depend on the duration of DM, which was less than ten years in most of our patients. Furthermore, the most frequently found abnormality in the upper limb was adhesive capsulitis involving the shoulder, which is in contrast to the existing data depicting hand involvement as the commonest [11,16,25].

According to international data, DCT is the most frequently (13–63%) seen abnormality among all the upper limb musculoskeletal complications [16,20,21]. However, we encountered a significantly low percentage of patients (1%) with DCT. It may be because that we have excluded diabetic subjects with chronic liver disease or alcoholism which are known confounding factors in the pathogenesis of DCT.

The results of our study indicate that there may not be a significant association of DCT with Asian diabetics. DCT appears to be an extremely common disorder affecting Caucasians of Northern European ancestry [26,27]. There is a possibility that the multi-factorial etiology of DCT has a strong environmental factor, based on the results produced by Finsen [28] in 2002 who found that family members were more likely to develop DCT if they were residing in the same geographical area as their affected relatives. In the same study, it is seen that although there is a high prevalence of DCT in Bosnia, there is a lower prevalence in the Bosnian Muslim community compared to the Serbian and Croat cohort, although a single definitive reason cannot be identified. This is very similar the case with our study and that of our neighboring country India where the majority is non-Muslim and the frequency of DCT is 28.75% compared to 1% in our study. The most important confounding factor is alcohol consumption as it can cause DCT independent to its affect on liver [29].

Muslims are prohibited to drink alcohol for religious reasons, which might be the cause of its low incidence in a Muslim community. Therefore, studies from Muslim majority regions are needed to further confirm or negate these results.

A study done in US [30] confirmed the incidence of adhesive capsulitis being two to four times higher in diabetics than in the general population and the prevalence of diabetes in patients with adhesive capsulitis was shown at 38.6%. Cagliero [16] in his study identified a frequency of 12% capsulitis in diabetic patients and although the duration of diabetes was shorter in our study, it is comparable to our study with a frequency of 11% . Both studies showed a predisposition of rheumatic complications in the female sex. Other studies done in UK, Australia and India show much higher frequencies of capsulitis ranging between 23–30% [17,20,21].

LJM is the commonest of all hand abnormalities found with a frequency of 9.5% which again is much lower than similar studies done in our region or internationally which vary between 20–50% [17,20,21]. A study done by Ardic [15] et al. showed similar results when they found none of their diabetic patients (n=78) with LJM, the duration of diabetes, however, in both the studies was less than ten years. Despite this difference in frequency, we found significant association with increasing age and duration of diabetes as in previous studies [4,5,31,32]. Trigger finger is as frequent in this study (i.e. 3.8%) as in India (3%) [21], Turkey (3.8%) [15] and USA (5%) [16] but lower when compared with Australia (11%) [20] and UK (29%) [17].

It is a well established fact that CTS is the commonest entrapment neuropathy involving one third of the diabetic population, however, this may be an underestimate since only one-sixth of the patients suffering from it report symptoms [13].

We observed a frequency of 9% of diabetic cases with CTS compared with 2% of the controls. Worldwide, the incidence of CTS in the diabetic population has consistently been reported as between 11% and 21%, in numerous studies [33-35] which is higher than what is observed in our study. Most of the patients with CTS in this study (i.e. 15 out of 19)belong to the obese category, which is a well recognized predisposing factor along with diabetes, although which of these has affected the frequency most remains questionable . Furthermore,genotype, environmental factors, lifestyle and occupation also play a role. Therefore, further randomized control trials are needed to understand these factors better and thus take measures to lower the incidence in more prevalent areas.

## Conclusion

Higher frequency of hand and shoulder involvement was observed in our diabetic population as compared to controls. Shoulder involvement is higher in our population than individual hand abnormalities as compared to international data. The duration of diabetes in our study was less than that observed in the existing data, which may be the reason of low percentages of complications observed in this study. It is recommended that diabetic patients should always be screened for the presence of rheumatic complications since early recognition lessens the chances of irreversible damage. Regular physiotherapy program should be the cornerstone of diabetic management along with diet and pharmacotherapy. The low frequency of DCT in our cohort, in the absence of alcoholism and liver disease, implies the presence of confounders in similar studies done previously thus further studies are needed to confirm or negate these observations.

## Abbreviations

DM: Diabetes Mellitus; BMI: Body Mass Index; GALS: gait, arm, legs, spine; REMS: Regional examination for musculoskeletal system; LJM: Limited Joint Mobility; CTS: Carpal Tunnel Syndrome; DCT: Dupuytren’s Contracture.

## Competing interests

Upon acceptance of the original article titled “Upper limb musculoskeletal abnormalities in Type 2 diabetic patients in a low socioeconomic strata in Pakistan” all copyright ownership for the manuscript is transferred to Biomed central research notes. The undersigned stipulate that the material submitted to the journal is new, original and has not been submitted to another publication for concurrent consideration. We also take the moral and ethical responsibility of not using unfair means such as plagiarism, duplicate publication, cross referencing, copying verbatim statements from other sources without acknowledgement in preparing this article. We have not received reimbursements, fees, funding, or salary from an organization that may in any way gain or lose financially from the publication of this manuscript, either now or in the future. We do not hold any stocks or shares in an organization that may in any way gain or lose financially from the publication of this manuscript. We are not currently applying for any patents relating to the content of the manuscript nor have we received reimbursements, fees, funding, or salary from an organization that holds or has applied for patents relating to the content of the manuscript. We further attest that we have not taken any financial support from any institution, nor there are any nonfinancial competing interest to declare in relation to this manuscript which could lead to a conflict of interest. We have not submitted the present article anywhere else.

## Authors’ contributions

SSK conceived the study, collected the data and drafted the manuscript. LW helped in analysis and interpretation of data. SAS revised the manuscript critically for important intellectual content. RMK performed the statistical analysis and helped in discussion. IG helped in drafting the manuscript. IMS did the critical analyses and corrections. All authors read and approved the final manuscript.
